# Genetic Gain in Yield and Associated Changes in Agronomic Traits in Wheat Cultivars Developed Between 1900 and 2016 for Irrigated Ecosystems of Northwestern Plain Zone of India

**DOI:** 10.3389/fpls.2021.719394

**Published:** 2021-09-23

**Authors:** Rajbir Yadav, Soma Gupta, Kiran B. Gaikwad, Naresh Kumar Bainsla, Manjeet Kumar, Prashanth Babu, Rihan Ansari, Narain Dhar, Palaparthi Dharmateja, Rajender Prasad

**Affiliations:** ^1^Division of Genetics, ICAR-Indian Agricultural Research Institute, New Delhi, India; ^2^ICAR-Indian Institute of Seed Science, Mau, India; ^3^Borlaug Institute for South Asia, Jabalpur, India; ^4^Department of Design of Experiment, ICAR-Indian Agricultural Research Institute, New Delhi, India

**Keywords:** bread wheat, genetic gain, mega cultivars, phenotypic plasticity, response to the competition

## Abstract

Knowledge about the yield gain over the years due to associated changes in the yield component traits is essential for a critical understanding of yield-limiting factors. To estimate genetic gain in grain yield (GY) and component agronomic traits of wheat varieties released between 1900 and 2016 for northwestern plain zone (NWPZ) of India and to identify agronomic and/or genetic basis of the realized gains, two sets of wheat varieties comprising mega varieties and two recently developed varieties were evaluated under timely sown, tilled, and early sown conservation agriculture (CA) conditions for four consecutive years under irrigated conditions. The average annual genetic gain in GY since 1,905 under timely sown irrigated conditions was found to be 0.544% yr^−1^ over the average of all varieties and 0.822% yr^−1^ (24.27 kg ha^−1^ yr^−1^) over the first released variety, NP4. The realized mean yield increased from 2,950 kg ha^−1^ of the variety NP4 released in 1,905–5,649 kg ha^−1^ of HD3086 released in 2014. Regression analysis revealed a linear reduction in height and peduncle length (PL) over the years with a simultaneous and linear increase in biomass at the rate of 43.9 kg ha^−1^ yr^−1^ or relatively at 0.368% yr^−1^ mainly because of delayed heading and increased crop duration. Regression analysis showed no linear trend for tiller number and thousand-grain weight (TGW). Though harvest index (HI) was found to linearly increase relatively at the rate of 0.198% per annum, polynomial regression improved the fitness of data with the indication of no increase in HI since 1982. Interestingly, genetic gain evaluation under early sown CA conditions for 4 years showed similar relative gain (RG) [a relative improvement in varieties across breeding periods (BP)] (0.544% yr^−1^) but with a higher absolute value (29.28 kg ha^−1^ yr^−1^). Major mega varieties like Kalyan Sona, HD2009, PBW 343, HD2967, and HD3086, which occupied a comparatively larger area, were found highly plastic to the improvements in the production environment under timely sown conditions.

## Introduction

Wheat (*Triticum aestivum* L.), being an important source of carbohydrates, protein, vitamins, and many other essential mineral elements, is the most widely grown crop in the world. Global wheat consumption is likely to be 834.8 MT by 2028 (OECD/FAO, 2019). Despite impressive growth in global wheat production in the recent past, it will be still challenging to meet this demand due to increased competition for alternate use of land, depleting natural resources, degrading soil health, biotic stresses, and changing climatic conditions (Yadav et al., 2010, 2017). To feed the still increasing world population, though, at an unequal rate in different parts of the world, the global average productivity of wheat has to be increased at the rate of 1.3% yr^−1^ (Rosegrant and Agcaoili, 2010). Stagnating productivity in Europe (Brisson et al., 2010) that relatively lowers gain in the high yielding environments (Maureen, 2019) and slowing yield gains in other countries (Fischer and Edmeades, 2010; Matus et al., 2012; Beche et al., 2014; Maureen, 2019) are worrying concerns. However, the recent estimates of genetic gain, the improvement in average phenotypic value due to selection within a population over cycles of breeding (Crespo-Herrera et al., 2017) for a mega wheat-growing environment in the northwestern plains of India are quite assuring. The difference in genetic gain realized across the world can be due to many factors including the available crop growth duration, agronomic practices followed, and prevailing weather and soil conditions. However, breeding improved varieties by nicking various agronomic traits has always been the main path to maximize the genetic gain through per unit area yield increments (Zhang et al., 2016; Gao et al., 2017; Mingliang et al., 2020).

Identification of yield-limiting factors is one of the crucial steps in designing any future breeding strategies. Genetic gains have largely been assessed by systematically evaluating the performance of historical varieties released over different points of breeding time (Sadras and Lawson, 2011; Sanchez-Garcia et al., 2013; Beche et al., 2014; Morgounov et al., 2014; Wu et al., 2014). Studies limited to a shorter period have reported more than 1% genetic gain per annum for wheat yield (Waddington et al., 1986; Sayre et al., 1997; Underdahl et al., 2008) whereas, gain assessment spread over a longer period (Perry and D'Antuono, 1989; Siddique et al., 1989; Royo et al., 2007) have reported around 0.5% gain annually. In winter wheat, maximum gain in yield (around 1%) was realized during 1960–2000 (Abeledo et al., 2003; Brancourt-Hulmel et al., 2003; Zhou et al., 2007). However, century-long analysis highly subdued the gain (Cox et al., 1988; Austin et al., 1989; Berzonsky and Lafever, 1993; Donmez et al., 2001). Since the beginning of the twenty-first century, progress in increasing the potential yield began to slow down in winter wheat or even reached to plateau in some of the countries (Barutçular et al., 2006; Peltonen-Sainio et al., 2009a; Graybosch and Peterson, 2010) and therefore, increased world wheat demand is most likely to be met by the South and East Asian countries including India. Periodic evaluation of released cultivars for associated changes in physiological and agronomic traits can provide the much-needed cue for future breeding strategies. In China, for example, grain weight and spike weight along with HI and biomass production were found to be the most exploited traits by wheat breeders since mid twentieth century (Zhou et al., 2007). In Australia, on the other hand, it was mainly the improvement of HI, which was responsible for yield gain (Sadras and Lawson, 2011). The increased spikes number (@ 0.30% yr^−1^) and grains per spike (GPS) (@ 0.60% yr^−1^) have mainly brought the yield gain in Spain (Sanchez-Garcia et al., 2012). A major jump in yield gain in the past has been realized with the introduction of dwarfing genes, which by reducing the size of vegetative plant organs resulted in better availability of assimilates to reproductive organs and thereby leading to higher yields through improved harvest index (HI) (Brancourt-Hulmel et al., 2003; Álvaro et al., 2008). Recent studies, however, indicate that future gain in yield will come through improved biomass by integrating modern genomic tools and a better understanding of physiological processes (Reynolds et al., 2011; Yadav et al., 2017) along with structural and agronomic adjustment for lodging resistance (Fischer and Edmeades, 2010; Yadav et al., 2017; Bainsla et al., 2018, 2020). India had a remarkable run of wheat production since 2011 with some intermittent hiccups due to climatic uncertainty. However, India cannot afford to be complacent on wheat production because of the likely increased demand due to a still-growing population and widening food security net by the Government of India. Wheat being central to food security in India is cultivated throughout the country; however, the northwestern plain zone (NWPZ) with about 11.59 Mha area, largely under irrigated condition (more than 95%), and accounting for 50% of Indian wheat production is the most important environment available in the country. It was, therefore felt necessary to examine the pattern of yield gain through changes in agronomic traits so that future breeding strategies can be formulated through the identification of important yield-limiting factors in modern cultivars.

High fluctuation in realized yield among many important states of northern India, such as Haryana, Rajasthan, Uttar Pradesh, and Bihar (Yadav et al., 2019) is frequently observed largely because of a sudden and abrupt rise in temperature toward the terminal growth stage of the crop, management constrains (Peltonen-Sainio et al., 2009a,b; Peltonen-Sainio and Hakala, 2011) and no time for compensation in short-growing varieties particularly under late-sown conditions. Trait plasticity, a response of different traits to environmental changes and an environmental contingent trait expression (Sadras and Rebetzke, 2013) have remained as important adaptive genetic traits and are highly relevant under changing climatic conditions. With no limitation of land in ancient agriculture, the success of agriculture was measured in terms of produce harvested from the quantity of seed sown and therefore, generally resulting in highly competitive plant type with a large sink and profuse tillering (Dewitt et al., 2004). Restricted availability of agricultural land for crop production has shifted the focus toward communal plant type, yielding more per unit of sown area. Besides this, the tradeoff between different yield contributing traits is the biggest stumbling block for future gain and for maximizing the fitness of genotype for better yield realization, the plasticity of different traits needs to be better understood (Peltonen-Sainio and Hakala, 2011).

In this study, we took the historical mega varieties, varieties covered the maximum growing area of their respective released time and recently released high yielding varieties for NWPZ, and breeding period (BP) for this study spanning over the last century and one and half decades of the current twenty-first century. Besides trait *per se* improvement, we were more interested to know the plant traits which were responsible for increment in plasticity resulting in their large-scale adaptation in Indian wheat-growing areas. We have also tried to analyze to know whether the recent gain in wheat yields was largely due to the development of communal or competitive genotypes by the wheat breeders.

## Materials and Methods

### Plant Material and Experimental Conditions

The experimental material consisted of two sets of bread wheat varieties (14 varieties in Experiment 1 and 10 varieties in Experiment 2). These varieties included the mega wheat varieties predominantly grown in NWPZ of India during the twentieth and in the first decade of the twenty-first century (1900–2016) and high yielding recently released varieties along with one variety registered with the protection of plant variety and farmer's rights authority (PPVFRA), Delhi, India for its suitability to conservation agriculture (CA) condition (CA, growing crop under zero tillage with previous crop residue retention). The list of varieties, their pedigree, developing institute, and the year of identification/release are detailed in Table 1. Experiment 1 was conducted under irrigated timely sown conditions, while, Experiment 2 was conducted in early sown irrigated CA condition. Experiment 2 was conducted to provide sufficient growth duration to long-duration varieties as in India we witness a high temperature toward the terminal stage of crop growth which forces each variety to mature fast. The varieties were chosen as they are assumed to cover the maximum wheat-grown area during their period of cultivation except for two recent varieties i.e., HDCSW 16 and HDCSW 18 in NWPZ (a prominent wheat-growing region with more than 50% contribution to the total wheat production of a country) of India since 1905 till 2016.

**Table 1:** List of genotypes, parentage information, developing Institute, and their year of release.

**Sl. No**.	**Varieties**	**Experiments**		**Parentage**	**Institute^#^**	**Year of release**	**Breeding period**
		**Timely Sown**	**Early sown**				
1.	NP4	✓	✓	SEL.HETEROZYGOUS LINE OF LOCAL MUNDIA	ICAR-IARI, New Delhi	1905	I
2.	C 591	✓	✓	Type9/8B	PAU, Ludhiana and HAU, Hisar	1934	
3.	C 306		✓	RGN/CSK3//2*C591/3/C217/NI4//C281	CCSHAU, Hisar	1965	II
4.	Sonora 64	✓		YT54/N10BII2*Y54 ORIGIN	CIMMYT, Mexico and PAU, Ludhiana	1965	
5.	Kalyan Sona	✓		PJ SIB/GB55	CIMMYT, Mexico and PAU, Ludhiana	1967	
6.	HD 2009	✓	✓	LR 64A /NAI 60	ICAR-IARI, New Delhi	1975	III
7.	WL 711	✓	✓	SKA/ CHR//KAL	PAU, Ludhiana	1977	
8.	HD 2329	✓	✓	HD 1962/E 4870/3/K 65/5/HD1553/4/UP262	ICAR-IARI, New Delhi	1985	IV
9.	WH 542	✓	✓	JUP /BJY”S”//URES	HAU, Hisar	1992	V
10.	PBW 343	✓	✓	ND/VG9144//KAL/BB/3/Y ACO'S' /4/VEE^#^5 ‘S	PAU, Ludhiana	1996	
11.	DBW 17	✓	✓	CMH79A.95/3*CNO 79//RAJ3777	ICAR-IIWBR, Karnal	2006	VI
12.	PBW 550	✓	✓	WH 594/RAJ 3856//W 485	PAU, Ludhiana	2007	
13.	HD 2967	✓	✓	ALD/CUC//URES/HD2160M/HD2278	ICAR-IARI, New Delhi	2011	VII
14.	HDCSW 16(Registered with PPVFRA for CA)	✓		CL1449/PBW343	ICAR-IARI, New Delhi	2012	
15.	HD 3086	✓	✓	DBW14/HD2733//HUW468	ICAR-IARI, New Delhi	2014	
16.	HDCSW18		✓	PBW 343/CL 1538	ICAR-IARI, New Delhi	2016	

# *ICAR-IARI-Indian Agricultural Research Institute, New Delhi, India; PAU-Punjab Agricultural University, Ludhiana India; ICAR-IIWBR-Indian Institute of Wheat and Barley Research, Karnal, India; HAU-Hisar Agricultural University, Hisar, India*.

### Field Design and Data Collection

The first experiment with 14 widely adapted bread wheat varieties was conducted during 2012–13, 2013–14, 2016–17, and 2018–19 crop seasons in the experimental farm of Indian Agricultural Research Institute (IARI), New Delhi under irrigated (5–6 irrigations, each at 20 days interval until physiological maturity to maintain optimal moisture conditions) timely sown conditions (≈ November 10). During 2012–13 and 2013–14, the material was raised in a randomized block design of two replications in a plot of six rows of 5.0 m length spaced at 0.20 m apart with a seeding density of 350–400 seed m^−2^. Excluding two border rows, all the data points were collected on only the central four rows, to avoid any border effects. During 2016–17 and 2018–19, the plot size was increased to 12 rows with similar spacing and row length, and data were collected on the central 10 rows keeping the same experimental conditions. A uniform population was maintained in each plot by seeding rate equivalent to 100 kg/ha for an approximate 36 g/1,000 seed test weight. The soil at the experimental site is alluvial with slightly alkaline characteristics and clay loam texture having low organic matter. The area has a semi-arid and sub-tropical climate with an average annual rainfall of 700 mm and the crop was irrigated as and when required. Fertilizer dose equivalent to 120 kg of N, 60 kg P_2_O_5_, 60 kg of K_2_O, 25 kg of ZnSO_4_ per ha was applied during 2012–13, 2013–14, and 2016–17 whereas, during 2018–19, the dose of nitrogenous fertilizer was increased to 150 kg ha^−1^ under conventional tillage. Half the dose of urea and a full dose of P_2_O_5_ and K_2_O were incorporated in the soil before seeding as basal fertilizers. The remaining half of the urea was applied as a top dressing after the first and second irrigation. As crop lodging has been a more common phenomenon in these years in India, a non-shading net was used to avoid the lodging of the crop under conventionally tilled conditions.

The second set of experiment was carried out with two assumptions: (i) that pregreen revolution cultivars in the absence of irrigation condition used to be sown in mid-October under conserved moisture conditions (Yadav et al., 2017), and their sowing in November might not give them an environment for their potential expression and, (ii) as the duration of the many high-yielding varieties have increased over the years, which exposes them to heat stress toward the terminal stage and therefore, might not be able to realize their full yield potential in November seeding (**Figures 6A–D**). To make the evaluation more competitive, we added C306, a variety released for early sown conditions with comparatively long duration, and replaced HDCSW 16 with HDCSW 18, a variety released for early sown conditions. Some of the other varieties like Kalyan Sona, Sonora 64, DBW 17, and WH542 were dropped due to their less strong or adverse response to early seeding. This experiment with 10 released varieties was carried out during 2015–16, 2016–17, 2017–18, and 2019–20 under CA condition by seeding around October 25, an early sowing condition for wheat in India. In this experiment, the dose of fertilizer was the same except for 120 kg N ha^−1^ during the first 2 years and 150 kg N ha^−1^ in the last 2 years, and the mode of fertilizer application was kept the same as in Experiment 1. Under the no-till condition, no support was provided with the non-shade net as CA generally supports high biomass without the incidence of lodging even for tall varieties. In the case of the experiment under CA conditions, glyphosate was used before seeding of wheat crop to kill all germinated weeds.

In both experiments, the incidence of wheat rusts and aphid infestation were controlled with a prophylactic spray of a fungicide, propiconazole 25 EC, and pesticide, imidacloprid @ 20 g active ingredient (a.i.) per ha. Weed infestation was controlled either manually or by the application of selective herbicide as per need arisen. In all growing seasons, efforts were made to create a non-yield-limiting environment. Both the border rows and the remaining four central rows were hand-harvested, threshed, dried, and weighed separately to record grain yield (GY) as tons ha^−1^ at a maximum of 12% seed moisture content. The traits, 50% days to heading (DH), days to physiological maturity of dry matter (DM), plant height (PH), ear length (EL), number of tillers, number of GPS, thousand-grain weight (TGW), peduncle length (PL), coleoptile length (CL), spikelets per spike (SPS), total biomass, and GY were measured in different trials. The data on DH and DM were recorded as per Zadocks stage 59 and Zadoks stage 89, respectively, when more than half of the tillers exhibited heads out and the day when more than half of the spikes in a plot showed yellowing (Zadok et al., 1974).

Plant height was recorded at the time of physiological maturity by measuring the length from the base of the plant to the tip of the main spike excluding awns. The number of florets was counted 5–10 days after anthesis on 10 randomly selected spikes as the number of florets differentiated. The GPS was counted after harvesting and threshing the 10 random spikes from each plot at maturity. After harvesting and threshing, a random sample was taken from each plot and 250 grains were counted, dried in an oven at 65°C for 48 h to measure TGW. The GY and plant biomass were measured after harvesting the central four rows and drying in the field for 4 days with due care to avoid the humidity at night.

### Statistical Analysis

Analysis of variance was performed by assuming the effect of varieties, replicate, year, and BP as a fixed effect, characters were taken as a random effect and the interactions of varieties with year, the BP for a particular trait as the mixed effect. The analysis was done using R software (RStudio Team, 2016). Linear, quadratic, and cubical polynomial regression equations were drawn to decipher the effect of the date of release by using the mean data over 4 years in both experiments. The linear equation *yi* = *a* + *bxi* + *e* was used to estimate the absolute or relative gain (RG) (%) for yield and its component traits, where, *yi* is the dependent variable (character mean value) and *xi* is the independent variable (year of variety release), *a* is the intercept, and *e* indicates the residual error. Genetic gain, the improvement in average phenotypic value due to selection within a population over cycles of breeding was calculated as a mean improvement in average yield throughout the BP.

A stepwise regression analysis was performed to further identify the key traits responding to yield realization using the backward regression approach by eliminating the least contributing factors. The varieties × environment interaction was estimated and elaborated using additive main effect and multiplicative interaction (AMMI) and genotype main effect and genotype by environment (GGE) interaction models. The best linear unbiased prediction (BLUP)-based factor analysis interaction was used to identify the pattern of gain in yield in the varieties released during the different BPs. All the multivariate analysis was done in R software using *metan* R-package (Olivoto et al., 2019; Package “metan”, 2020). The correlation and principal component analysis (PCA) were also performed using R-software using ggcorplot (Kassambara, 2019) and factoextra (Kassambara and Mundt, 2019) packages.

### Phenotypic Plasticity and Response to Competition

To calculate phenotypic or trait plasticity separately in Experiment 1, each trait and variety was quantified by calculating the ratio of the standard deviation of the trait for each variety to the overall phenotypic standard deviation of the population of varieties. Response to competition (RC), which is the ability of plants to respond to available space, was calculated as per the procedure mentioned in earlier publications (Reynolds et al., 1994; Slafer and Savin, 1994).

## Results

### Analysis of Variance and Differences Among Wheat Varieties Released in Different Decades

Analysis of variance revealed significant differences between environment (year), varieties released in different years, and the BPs in India for most of the agronomic traits across years under testing for Experiment 1. The varieties were highly significant for all the phenological and component traits except for the number of tillers (Supplementary Table 1). Similarly, the year or environment was also significant for all the traits except GPS, PL, and CL. The variety × year was non-significant for yield, the number of tillers, and TGW and significant was for all other traits. The contribution of the BP toward the genotypic sum of squares was the largest for yield, biomass, PH, PL, and CL, and quite large for DH, DM, and TGW. The BP could significantly explain the variation in all the traits except the number of tillers. The BP × year was significant for biomass, PH, PL, and CL. The varieties within the period were found significant for DM, TGW, GPS, PH, SPS, and CL.

### Differences Among Cultivars for Yield, Biomass, and Harvest Index

**Figure 1:**
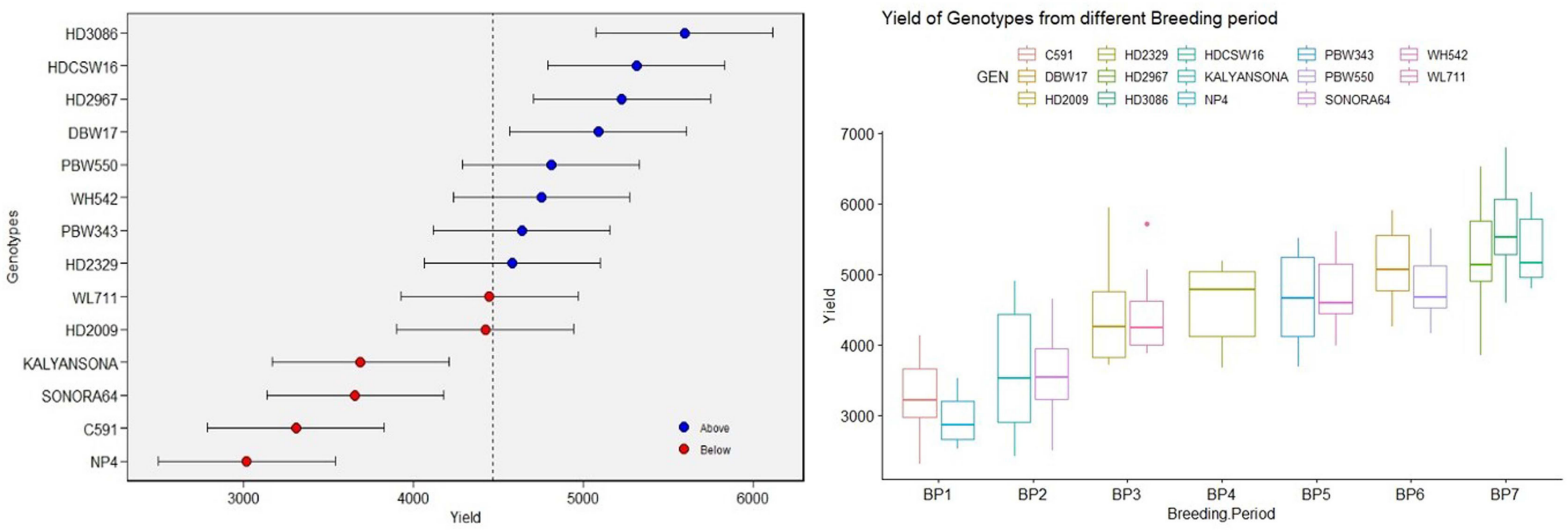
The box plot [based on the best linear unbiased prediction (BLUP)] of yield over varieties under timely sown conditions and yield of varieties from different breeding periods.

Yield averaged over 4 years showed consistent improvement since the beginning of the twentieth century when the initial variety NP4 was released for cultivation. The BLUPs estimated over 4 years of replicated GY data also indicated the same trend (Figure 1). The average GY of 2,949 and 3,253 kg ha^−^1, respectively, of the varieties NP4 (1905) and C591(1936) bred in the first breeding period (BP-I, Table 1) shows a remarkable gain in yield within the BP-I representing pregreen revolution breeding activities. The next gain was brought by the introduction of dwarf wheat varieties like Sonora 64 (3,618 kg ha^−1^) and Kalyan Sona (3,650 kg ha^−1^), representing the second breeding period (BP-II), and the gain in GY was equal to what we observed from NP4 to C 591. However, a quantum jump in productivity potential was brought about by indigenously bred wheat varieties like HD 2009 (4,419 kg ha^−1^) and WL 711 (4,446 kg ha^−1^), representing the third breeding period (BP-III). In the next decade and during the fourth breeding period (BP-IV), the variety HD 2329 was released for cultivation, whose average productivity realized in our experiments was 4,586 kg ha^−1^. This variety was highly tolerant to lodging because of its comparatively short stature and solid stem at the base. The lodging resistance provided opportunities to the farmers for increasing fertilizer application and realizing better yield. HD 2329 became the first indigenously bred wheat mega varieties occupying a very large area in NWPZ. In the subsequent years representing the fifth breeding period (BP-V), HD 2329 was replaced by Veery lines (IBL/IRS translocation) like PBW 343 and WH 542 with nominal gain in the yield. The yield gain of PBW 343 and WH 542 over HD 2329 was more apparent during 2018–19 when the urea application was increased to 150 kg ha^−1^. PBW 343 and WH 542 are of comparatively longer duration and their yield gain is probably more reflected in the crop year with the comparatively longer winter season. Farmers of Haryana and Punjab states in NWPZ realized a quantum jump in yield by seeding PBW 343 at the end of October or the first week of November against the middle of November seeding practiced for HD 2329. Varieties PBW 550 and DBW 17 released during the sixth breeding period (BP-VI) were not able to completely replace PBW 343 and WH 542, thus giving a scope for the next quantum jump. The biggest gain in GY was thus realized through recent mega varieties like HD 2967 and HD 3086 released during the seventh breeding period (BP-VII), which simultaneously replaced three varieties namely PBW 343, DBW 17, and PBW 550. Across varieties, biomass has ranged between 8,766 kg ha^−1^in NP4 to 14,235 kg ha^−1^in HDCSW 16 and it has increased linearly over the years at the rate of 43.60 kg ha^−1^ yr^−1^.

### Correlation, Principal Component, and Varieties × Environment Analysis

**Figure 2:**
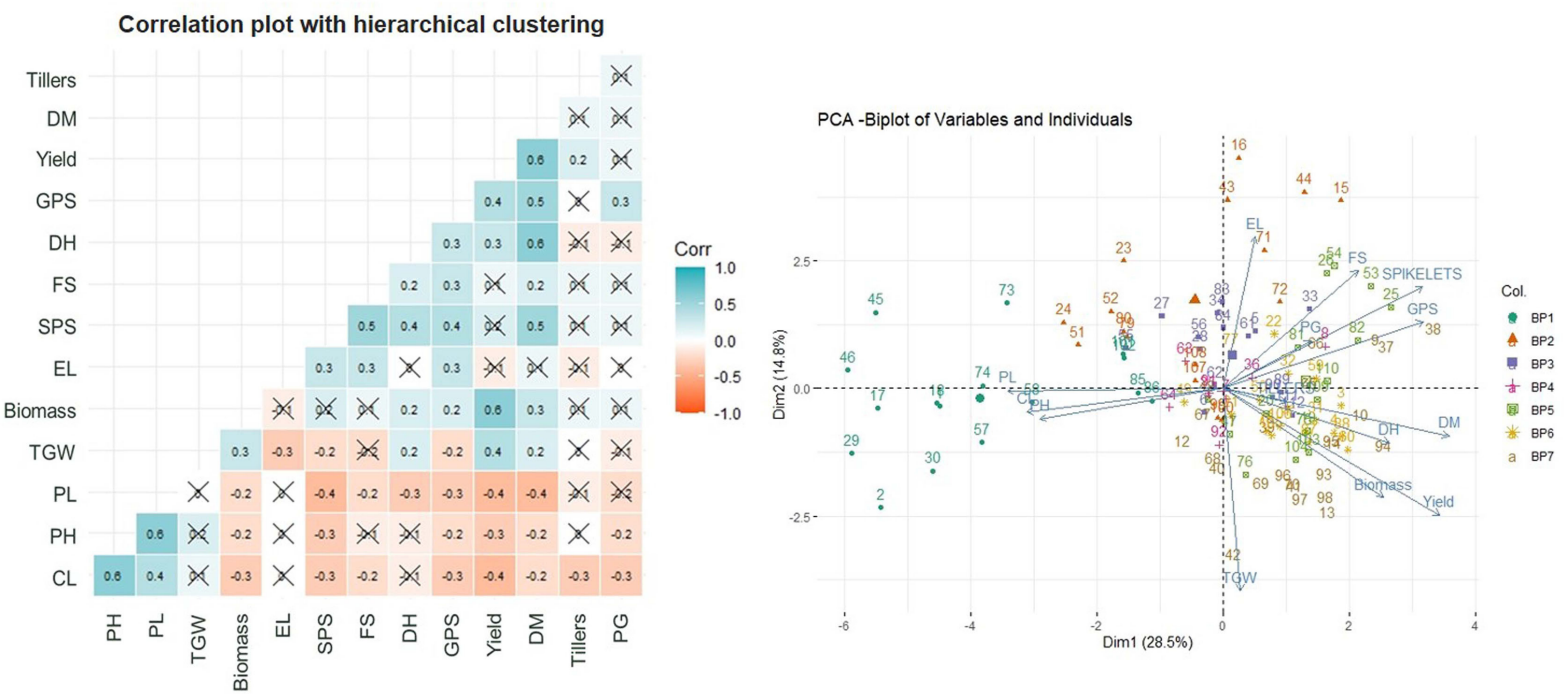
Correlation plot with hierarchical clustering of traits and principal component analysis (PCA).

The Pearson's correlation coefficients (Figure 2) between the traits under study indicated a very strong positive correlation of yield with DM and biomass followed by TGW, DH, and GPS. The negative correlation of the yield was observed with PL, PH, and CL.

The acute angle between the Eigenvector of yield with DM, biomass, and DH and also between themselves, indicate the relatedness in these traits through PCA (Figure 2). The TGW and GPS though have an acute angle of Eigenvectors with yield but have an obtuse angle with each other indicating exclusiveness of these traits. The data points of varieties bred in a given BP are largely following the grouping within the BP with some overlapping from BP-V to BP-VII.

The AMMI analysis showed a significant genotype and G × E interaction for GY (Supplementary Table 2). The AMMI biplot analysis could differentiate among the environments and the varieties as it combines the analysis of variance of genotypes and the environment main effects with PCA of the genotype environment interaction (GEI) into a unified approach.

### Regression Analysis for Yield and Other Agronomic Traits

The multiple regression analysis models identified DH, DM, TGW, PH, GPS, and biomass as highly significant traits while the number of tillers and CL were found non-significant. BP as a variable explained the larger amount of variance with an *R*^2^-value of 69% largely because of compounded inclusion effect of DH, PH, biomass, EL, and number tillers. The stepwise regression analysis (Supplementary Table 3) suggested for DM, biomass, TGW, CL, GPS, PH, and EL traits and thus explaining variance up to the tune of 65%.

**Figure 3:**
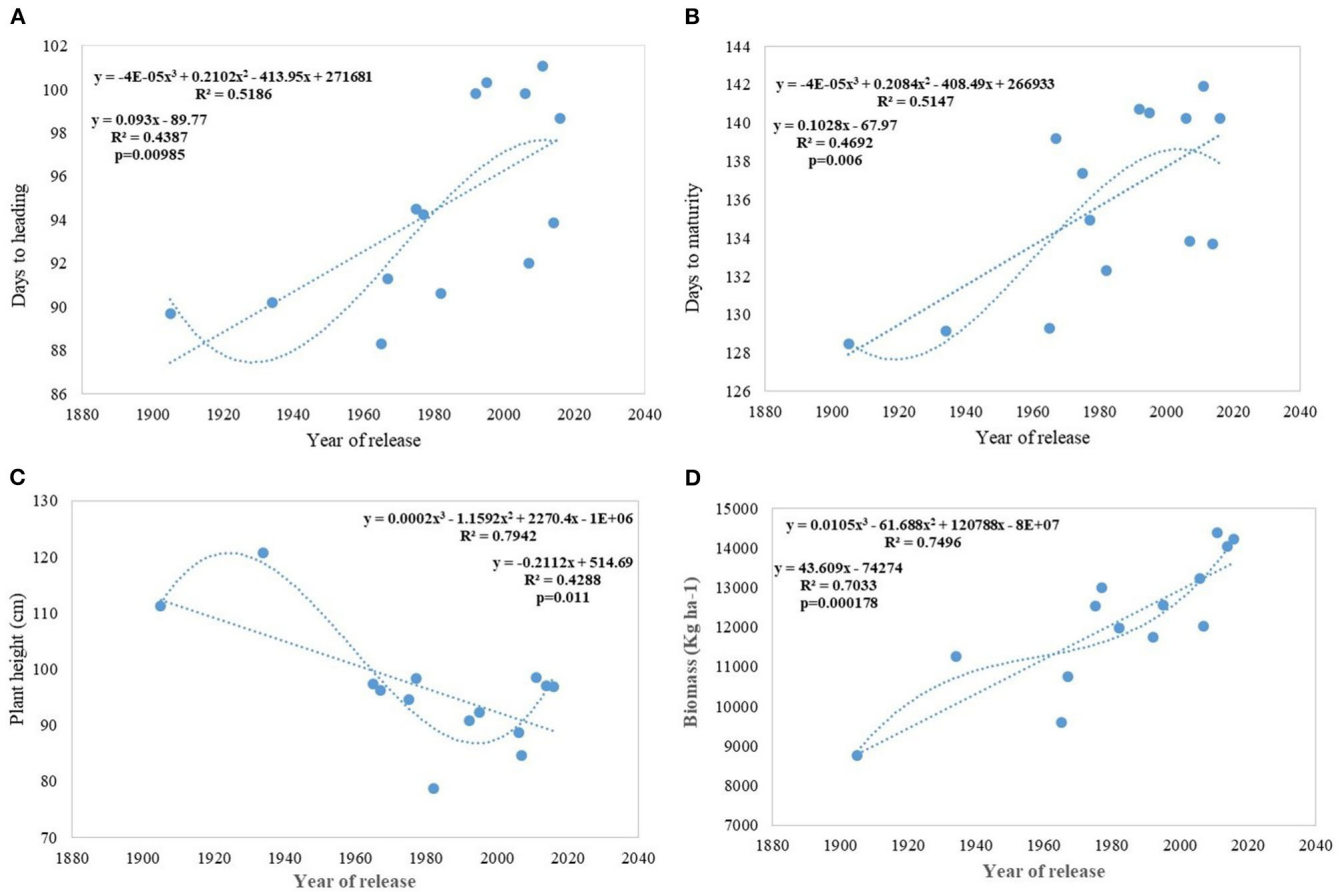
Regression equation between phenological traits and yield component traits against the year of release. **(A)** Days to heading, **(B)** Days to maturity, **(C)** Plant height (cm), **(D)** Biomass (Kg ha^−1^).

Analysis of regression detected a significant association between phenological stages and the year of release. Slopes of DH and DM vs. year of release were significantly >0 (Figures 3A,B). Association was much stronger between DM vs. year of release than with DH. The DH and DM have increased linearly since 1905 in Indian wheat cultivars. In the last 100 years, mean heading has been delayed at the rate of 0.09 days per year or 0.09% per year and the recent varieties head 4–12 days later than the initial variety, NP4. On the other hand, crop duration increased linearly at the rate of 0.010 days per year delaying the maturity by 5–13 days in the recent varieties. Polynomial regression shows a declining trend in both DH and DM after HD 2967, released in 2011. Both of these traits, however, are highly influenced by the environment and the interaction of varieties × year was very strong.

**Figure 4:**
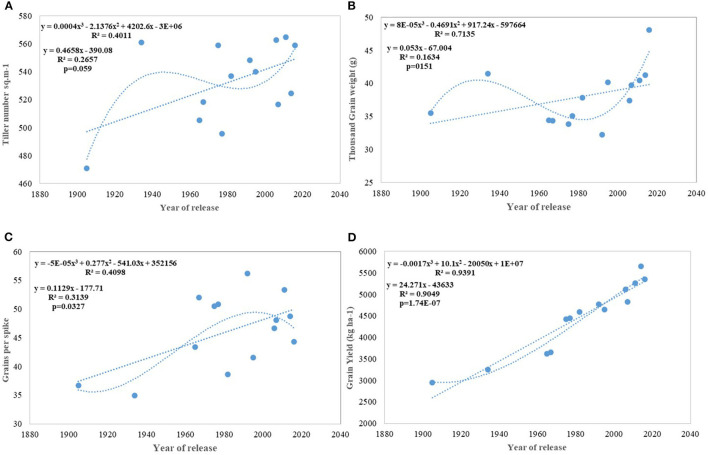
Regression equation between yield and its component traits with the year of release of wheat cultivars. **(A)** Tiller number per sq m^−1^, **(B)** Thousand grain weight (g), **(C)** Grains per spike, **(D)** Grain yield (Kg ha^−1^).

**Figure 5:**
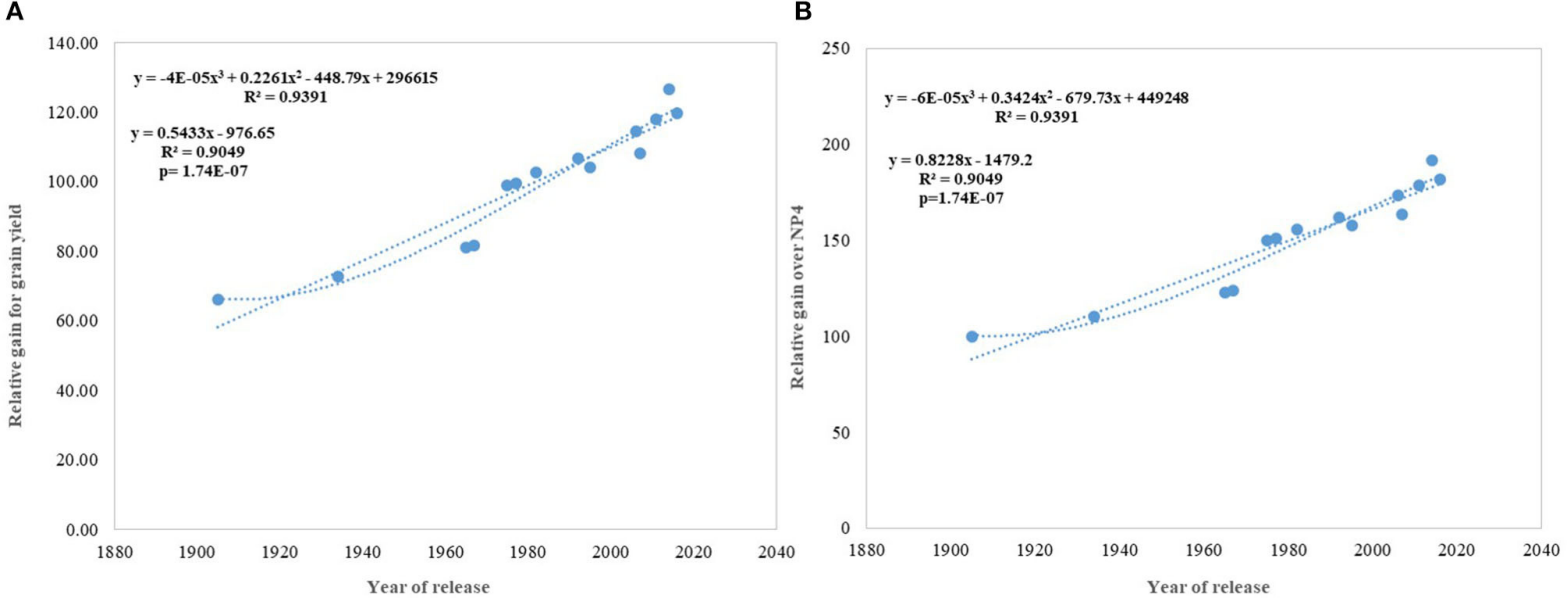
Relative genetic gain over the average of all varieties and NP4 as a base variety. **(A)** Relative gain for grain yield, **(B)** Relative gain over NP4.

Regression analysis showed that the year of release accounted for 90% of the variation in mean gain yield data of varieties released in different years and the increase in yield was linear over the years (Figure 4D). An absolute genetic gain for GY in the last 115 years is around 24.27 kg ha^−1^. Relative genetic gain has been found around 0.544% per year (over the average of all varieties) (Figure 5A) and 0.822% per year if calculated by taking NP4 as a base (Figure 5B). PH (Figure 3C) was also affected by both varieties and year; however, there was no significant varieties × year interaction. plant height ranged from 79 cm for HD 2329 to 121 cm of C 591 and the linear regression revealed a linear reduction in height with the year (*p* = 0.005); however, the cubical polynomial regression indicating increase in height of recent varieties significantly improved the relationship with *R*^2^-value as high as 0.7942.

Results for productive tillers m^−2^ shows that there has been a continuous linear improvement in spike m^−2^; however at a very slow pace of 0.46 tillers per year and with comparatively larger standard error (±25.78 tillers) (Figure 4A). Similarly, GPS (Figure 4C) also showed a linear increase over years at the rate of 0.112 GPS (*p* = 0.037) with the RG being 0.245% in the last 115 years. Flowering nodes have no linear relationship; however, polynomial regression shows that the flowering nodes first increase and then slightly drops. Interestingly, TGW (Figure 4B) showed no linear relationship but cubical polynomial significantly improved the relationship between TGW and the year of release (*p* = 0.01). In a curvilinear relationship, the year of release accounted for 71.3% of the variation in TGW.

The relative annual rate of growth for biomass was 0.358%. Like GY, the biomass production also does not indicate any saturation and the biggest gain (0.737) was realized after 1992 (WH 542). It was interesting to note that, both linear and cubical polynomial showed a significant relationship between biomass (Figure 3D) and the year of release. The HI has also increased linearly with the year of release from 0.35 in NP4 and 0.29 in C 591 to 0.41 in WH 542 released in 1992 and since then, there is no improvement in HI. The absolute and RG per year for HI was found to be 0.0007 and 0.373%.

### Genetic Gain Under the Early Sown Condition

**Figure 6:**
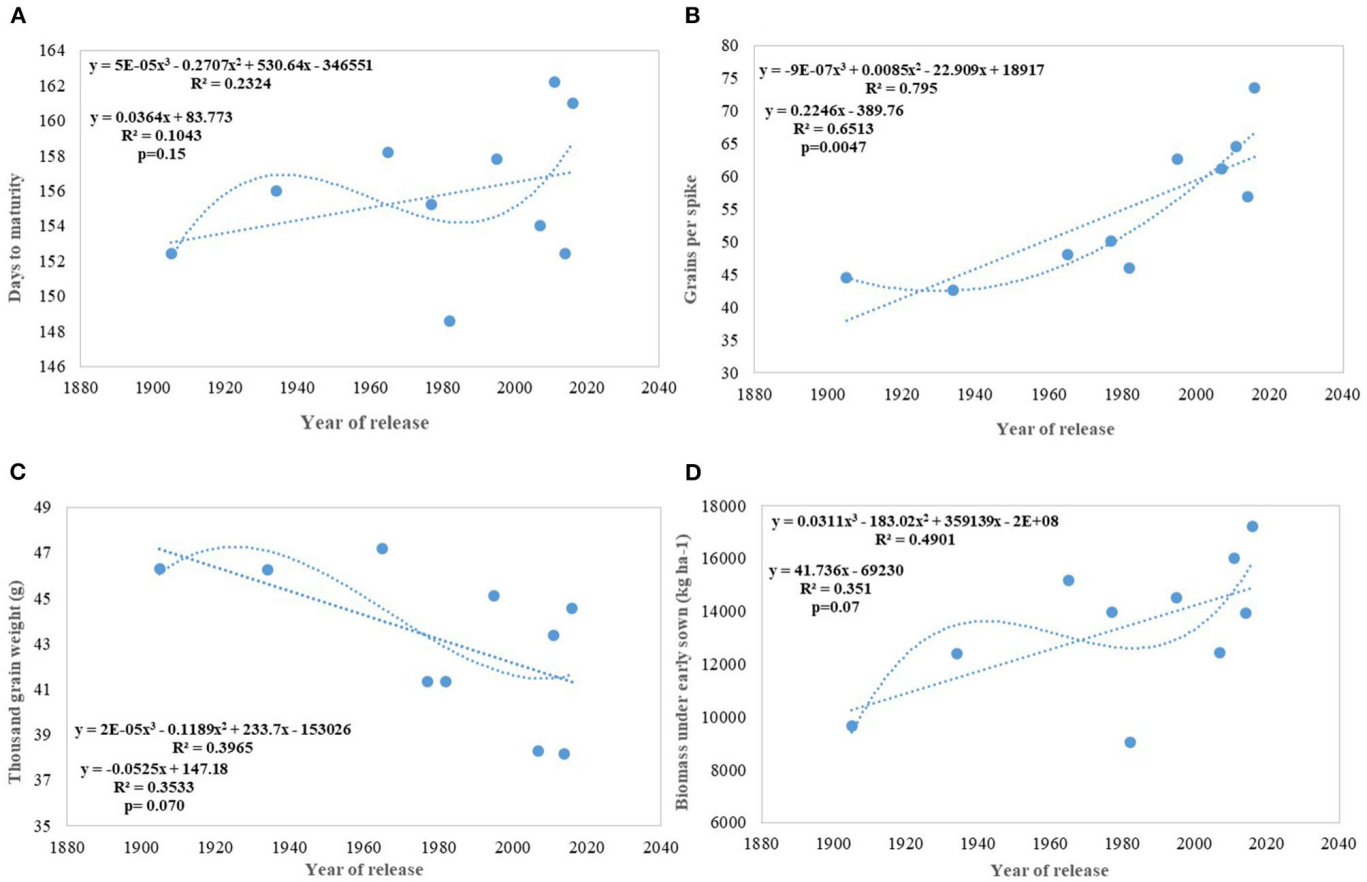
Regression equation between phenological and yield component traits against the year of release under early sown conditions. **(A)** Days to maturity, **(B)** Grains per spike, **(C)** Thousand grain weight (g), **(D)** Biomass under early sown (Kg ha^−1^).

**Figure 7:**
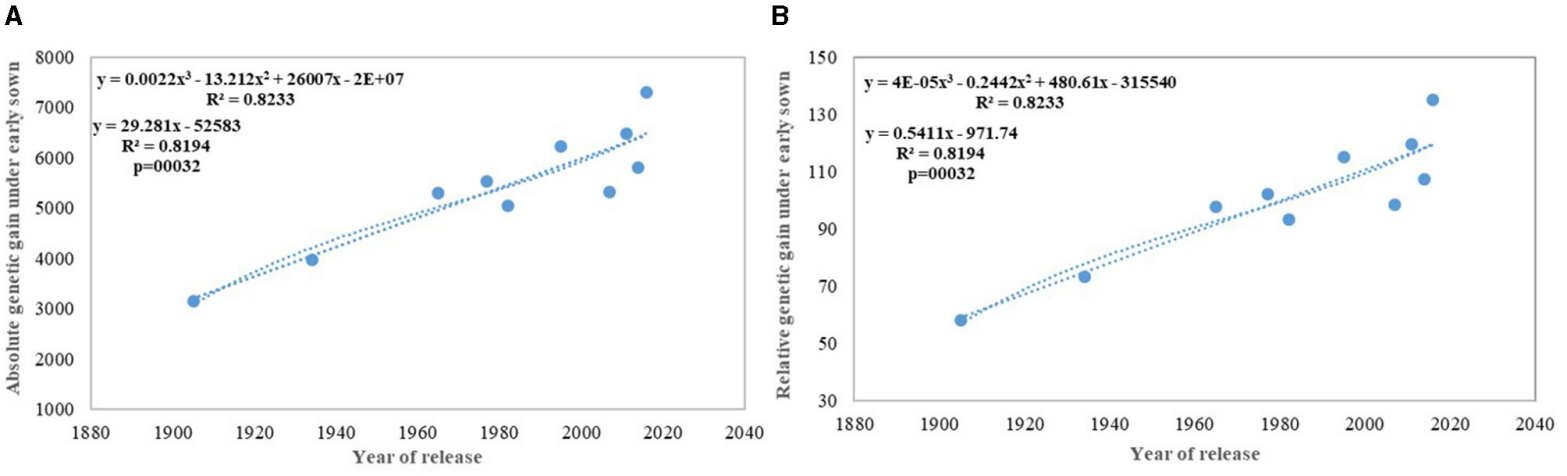
Absolute **(A)** and relative **(B)** genetic gain under early sown conditions.

We carried out this experiment with two assumptions: (i) that pregreen revolution cultivars in the absence of irrigation condition used to be sown in mid-October under conserved moisture conditions (Yadav et al., 2017), and their sowing in November might not give them an environment for their potential expression and (ii) as the duration of the many high yielding varieties have increased over the years, which exposes them to heat stress toward the terminal stage and therefore, might not be able to realize their full yield potential in November seeding (Figures 6A–D). To make the evaluation more competitive, we added C 306, a variety released for early sown rainfed conditions, and replaced HDCSW16 with HDCSW18, a variety released for early sown conditions. Some of the other varieties like Kalyan Sona, Sonora 64, DBW17, and WH542 were dropped. The absolute genetic gain realized in the experiment from 1905 till 2016 was 29.28 kg ha^−1^ yr^−1^, at least, 5 kg higher than that realized under timely sown conditions. However, the relative genetic gain measured over average value was almost similar in both sets of the experiment. The trend for a linear increase in the biomass is stronger under early sown conditions (Figure 6D) and seems to be a major factor besides the GPS for yield gain (Figure 6B). The TGW which was showing a slight increasing trend in timely sown condition clearly shows a declining trend in early sown condition (Figure 6C), mainly because the GPS in the varieties requiring mild vernalization is strongly increased leading to some compromise in TGW due to interfloret competition. The duration of stem elongation phase under the early sown condition in mild vernalization requiring variety is prolonged, resulting in a higher GPS. The absolute (Figure 7A) and relative genetic gain (Figure 7B) over the years showed a significant relationship with the GY and the year of release under early sown conditions.

### Response to Competition and Trait Plasticity

**Figure 8:**
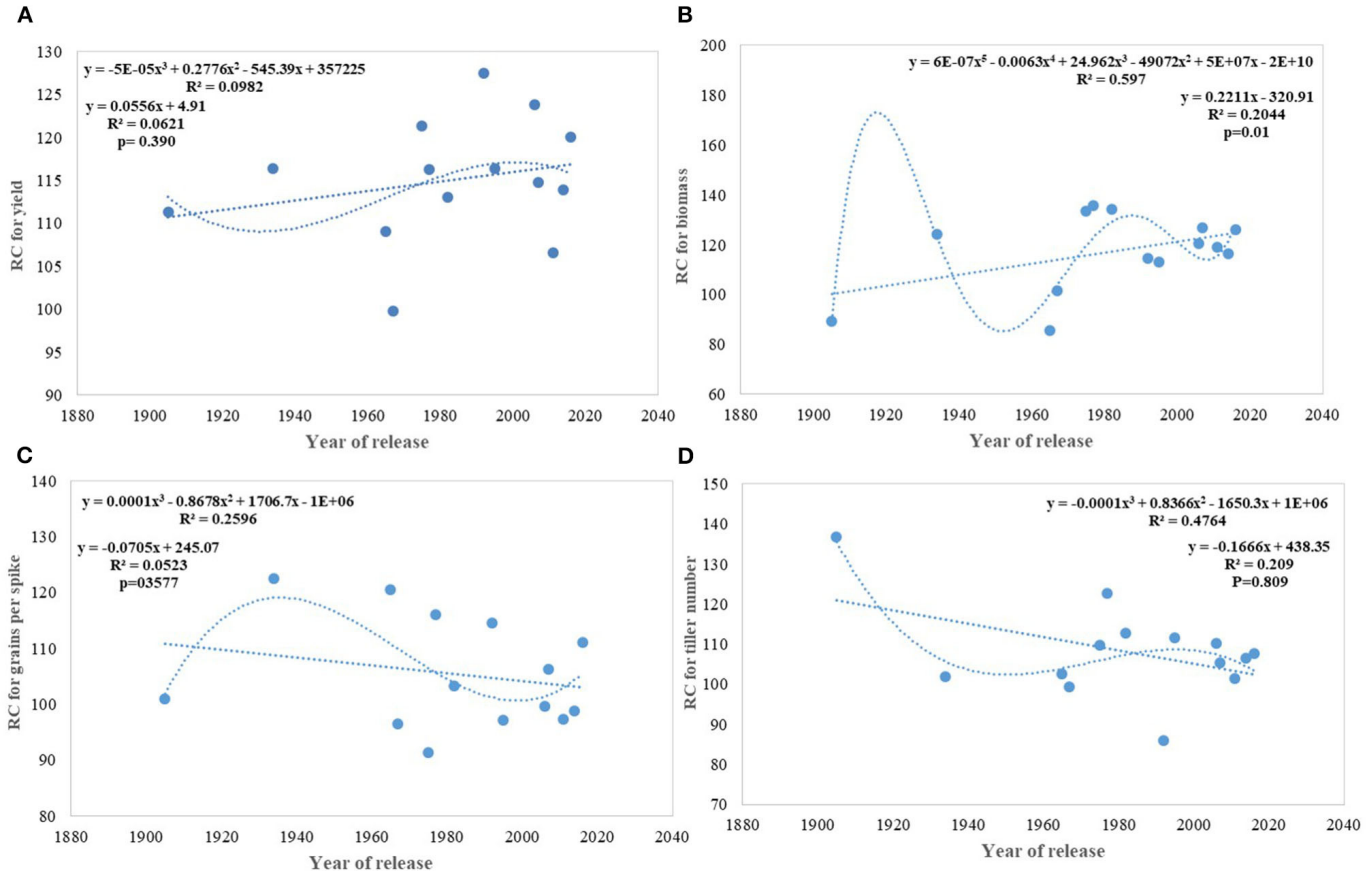
Relative competitiveness for yield and component traits against the year of release. **(A)** yield, **(B)** biomass, **(C)** grains per spike, **(D)** tiller number.

On average, border rows yielded 15% more than central rows of the plot largely because of more space availability between two adjacent plots. No trend was observed for the RC in the released cultivars for GY (Figure 8A). However, among the early cultivars, C 591 was highly competitive and dwarf wheat introductions were highly non-competitive for yield. Comparatively broader leaves in WL 711 and HDCSW 16, earlier canopy cover in PBW 550, compact plant type in HD 2329, and more ground cover in WH 542, and DBW 17 were probably providing them with a competitive advantage. In the present experiment, the response to the competition was calculated to assess whether we are moving toward communal varieties or not (Table 2). Though there is no trend, most of the dwarf varieties are showing comparatively lesser RC for GY. There is no linear trend for any of the component traits for RC except for biomass and tiller number (Figures 8B–D). Compilation and analysis of information related to the plasticity of various GY forming traits can enhance our understanding of why a particular cultivar becomes a mega cultivar. In Indian wheat cultivation history, probably four real mega cultivars can be defined during different period and these are Kalyan Sona in the 70s, HD 2329 in the 80s, PBW 343 in the 1990s, and now HD 2967 and HD 3086 in the second decade of the twenty-first century. All these cultivars except HD 2329 have comparatively higher GY plasticity. Plasticity has come from different traits in different cultivars i.e., from biomass, tiller number, and grain number in Kalyan Sona, from grain weight and grain number in PBW 343, and GPS in HD 2967 (Table 3). Overall, important varieties like Kalyan Sona, HD2009, HD2967, HD3086, and PBW 343 which occupied a comparatively larger area among the mega varieties, are highly plastic and are able to respond well to the improvement in the production environment.

**Table 2:** Grain yield and response to competition for yield and biomass in cultivars released over the years.

**Variety**	**Grain Yield (Kg/ha)**	**Response to competition**		**Type of genotype**
		**Yield**	**Biomass**	
NP4	2949.753	111.2949	89.41154	NC
C 591	3253.533	116.3677	124.3056	C
Sonora 64	3618.616	109.0458	85.70714	NC
Kalyan Sona	3650.995	99.79836	101.6185	NC
HD 2009	4419.811	121.3589	133.5878	C
WL 711	4446.363	116.2602	135.835	C
HD 2329	4586.856	113.029	134.3564	C
WH 542	4767.799	127.4854	114.5005	C
PBW 343	4646.559	116.4033	113.1505	NC
PBW550	5116.868	123.8471	120.5556	C
DBW 17	4825.611	114.7973	126.9841	C
HD 2967	5263.288	106.5891	118.9499	NC
HD 3086	5648.943	113.8737	116.5461	NC
HDCSW 16	5352.087	120.0304	126.2535	C

* NC, non competitive; C, competitive.

**Table 3:** Trait plasticity in the genotypes released over the last 100 years.

**Variety**	**Release year**	**Trait plasticity**			
		**Yield**	**Thousand grain weight**	**Grain number**	**Tillers number**
NP4	1905	0.383026	0.478035	0.276739	1.091124
C 591	1934	0.60013	0.247056	0.725794	0.922902
Sonora 64	1965	0.72137	0.23232	0.457019	1.327552
Kalyan Sona	1967	0.965657	1.292664	1.156357	1.240012
HD 2009	1975	0.743617	0.449985	0.215656	1.113342
WL 711	1977	0.636134	0.725085	1.231849	0.687181
HD 2329	1985	0.565496	0.988142	0.613168	0.813527
WH 542	1992	0.57611	0.264158	0.516264	1.004723
PBW 343	1996	0.67475	0.546696	0.619719	0.908506
PBW 550	2006	0.510284	0.552944	0.674058	0.787816
DBW 17	2007	0.55182	0.498022	0.582023	1.232777
HD 2967	2011	0.835045	0.504635	0.740293	0.612293
HD 3086	2016	0.74585	0.497173	0.922228	1.108953
HDCSW 16	2014	0.507393	0.577998	0.501025	0.775689

## Discussion

Our study, like many other earlier studies (Sanchez-Garcia et al., 2013; Zhang et al., 2016), shows a significant difference in GY and related yield component traits in the varieties released in different years and BPs. Having each BP represented by at least one variety, a variance analysis revealed little or no differences among the varieties within each BP for yield, biomass, tillers per plant, and the number of spikelets. Varieties within the BP were though differing for DM, PH, CL, GPS, and TGW indicating that no single phenological criterion was adopted by the breeders for developing varieties in a specific BP.

It is a common perception among the breeders of the current era that yield gain in wheat started with the introduction of dwarf varieties in 1964–66; however, the yield of C 591 over NP4 and also of C 306 in early sown experiment refutes this assumption and shows a remarkable gain within the first BP, representing the potential impact of pregreen revolution breeding activities. Current analysis shows that the next quantum jump in productivity potential was brought about by the release of the first-generation indigenous breeding products like WL 711 and HD 2329 by involving dwarf introduction in the crossing. Yield plateau imposed by HD2329, which remained dominating for 10–15 years was subsequently broken by the release of Veery lines like PBW 343 and WH 542 with IB/IR translocation. Farmers in the two major states of NWPZ amplified the realized gain by seeding PBW 343 slightly early because of its mild vernalization requirement and using a slightly higher dose of nitrogenous fertilizer. PBW 343 comprising *Yr23* and *Yr9* genes for yellow rust resistance became susceptible to yellow rust pathotype 78S84 and incidence and losses due to yellow rust started building up in the first decade of the twenty-first century. Subsequent releases like DBW 17 and PBW 550, could not completely replace these varieties. The formal release of HD 2967 in 2011 was a great relief to the farmers and within a short span of 3–4 years, it simultaneously replaced PBW 343, PBW 550, and DBW 17. HD 2967 became immensely popular among the farmers with its area crossing over 10 M ha in the Northern Plains and received unprecedented breeder seeds indents of 4,000 q for the year 2019–20 (ICAR-IIWBR, 2019). Yield gain in the Northern Plains was further consolidated by the subsequent release of HD 3086.

In contrast to studies on winter wheat (Barutçular et al., 2006; Peltonen-Sainio et al., 2009a; Graybosch and Peterson, 2010) and many other studies on spring wheat (Fischer and Edmeades, 2010; Matus et al., 2012; Beche et al., 2014; Maureen, 2019), where from the beginning of the twenty-first century, yield gain has either started to slow down or even plateaued, we found a strong linear increase in wheat yield since 1905 with no indication of yield saturation (Figures 4D, 5A,B). An absolute genetic gain of 24.27 kg ha^−1^ y^−1^ with a relative value of around 0.544% per year in a century-long period is equal or better than many other breeding programs in the world (Fischer and Edmeades, 2010; Sadras and Lawson, 2011). Early sown experiment revealed a comparatively higher absolute gain of 29.28 kg ha^−1^ yr^−1^ (Figure 6D) but with a similar relative value (0.544 %).

Regression analysis indicates DM and DH (Figures 3A,B) in Indian wheat varieties have linearly increased over the years significantly. This is in contrast to earlier reports that the yield gain in the Mediterranean environment has been due to earlier heading and other studies showing no chronological trend (Slafer et al., 2009). Analysis of these varieties for physiological traits like chlorophyll content (Gupta et al., 2017) shows no change or chronological trend and higher capturing of radiation due to increased duration rather than improved radiation use efficiency (Shearman et al., 2005; Sadras and Lawson, 2011) is the major reason for the increased biomass and yield gain. Linear increasing trend for leaf area index (LAI) along with near-perfect LAI 5.94 with semi-erect leaves observed in all-time mega variety HD 2967 (Gupta et al., 2017) indicated the importance of physiological traits, plant architecture, and crop canopy beside yield component in yield maximization. Polynomial regression fitting quadratic function of crop duration vs. year of variety release shows a declining trend in both DH and DM after the release of HD 2967 in 2011. Increased duration, however, introduced instability in the production over the years due to sudden rise in temperature toward the terminal stages (Yadav et al., 2019), and therefore further increase in the duration without adjusting the sowing time will be highly unrewarding. Highly significant environment and varieties × year interaction for both of these traits (DH and DM) indicated the same. The higher realization of yield gain in the early sown experiment was also largely because of the longer duration available to all varieties along with delay in the heading because of optimum combination of vernalization alleles with no reduction in grain filling duration in the adapted varieties. It is also realized that the weak linear trend for the increased duration and delayed heading (DH) in the early sown experiment was because of the presence of both mild vernalizations requiring and non-vernalization requiring genotypes in the study. However, still, DH accounted for 54% of yield variation in the early sown experiment and interestingly polynomial regression shows no saturation of relationship between delayed DH and higher yield.

**Figure 9:**
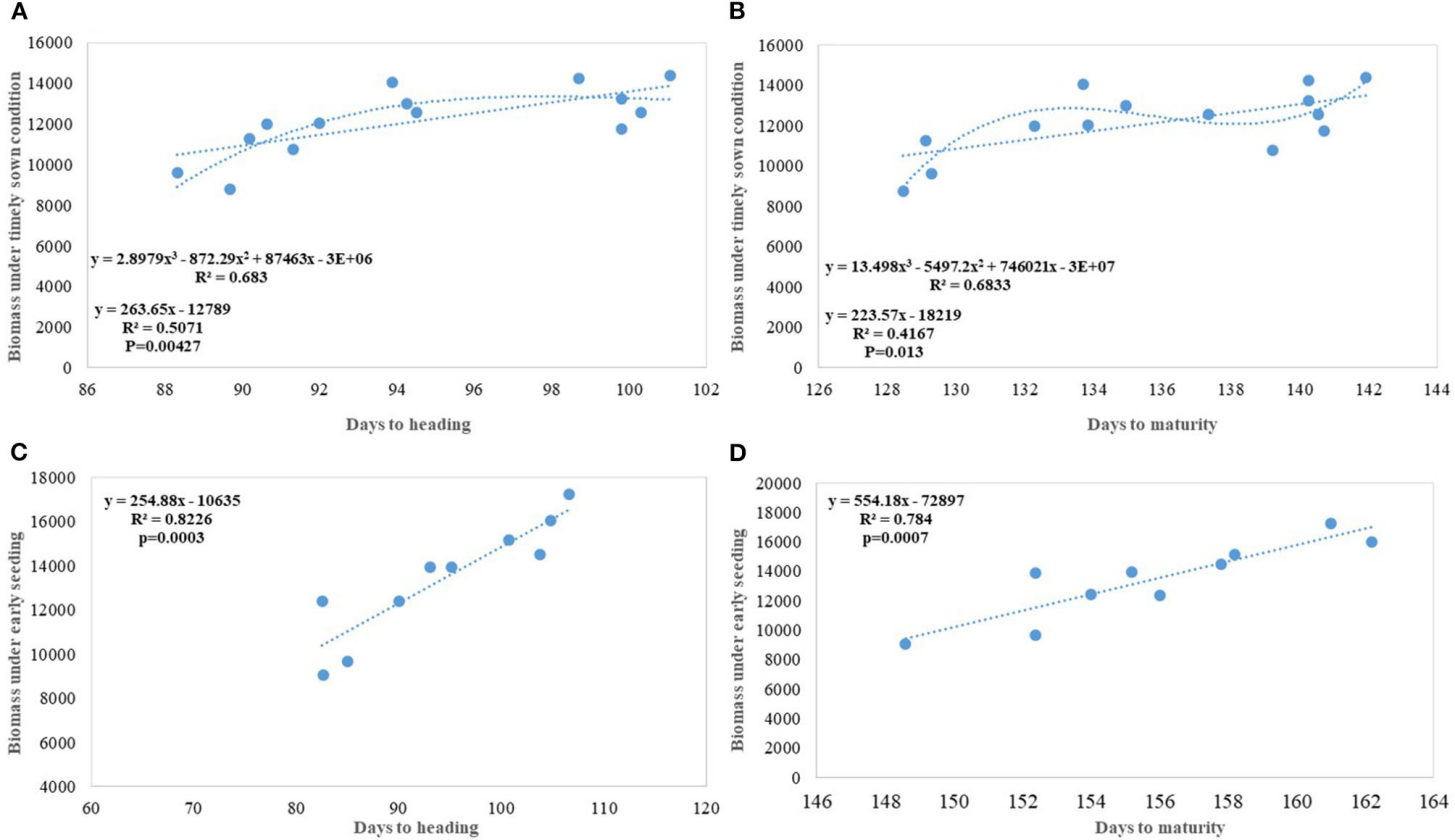
Increase in biomass due to increased duration and delayed heading **(A)** Days to heading (DH) vs. biomass under timely sown condition; **(B)** Days to maturity vs. biomass under timely sown condition; **(C)** DH vs. biomass under early seeding; **(D)** Days to maturity vs. biomass under early seeding.

Biomass has been one of the most important parameters influenced by wheat breeding activities in India during the twentieth and twenty-first century as the year of release alone explained 70% (*p* = 0.000178) of biomass variation in the released varieties tested under timely sown condition. Biomass has increased linearly at the rate of 43.60 kg ha^−1^ yr^−1^ in century-long analysis; however, fractured analysis shows the biggest gain (97.12 kg ha^−1^ yr^−1^) after 1992. As discussed in the earlier section, biomass-like GY increased linearly over the years due to a linear increase in the duration along with DH. Delayed heading explained comparatively more variation in biomass than days to maturity, probably because of the conflict introduced by forced maturity in longer duration varieties (Figures 9A,B). Quadratic equation fitted for both phenological traits vs. biomass, not only improved the level of fitness but also explained more variation.

In earlier studies, it was reported that biomass has increased due to improved photosynthesis, better stomatal conductance, higher leaf chlorophyll content (Maureen, 2019), and improved radiation-use efficiency (Bustos et al., 2013); however, in our earlier study, we found no trend for total leaf chlorophyll content and declining trend for stomata number (Gupta et al., 2016). In the present study, an increase in the biomass has been largely attributed to increased duration with DH (Figures 9A–D) and to some extent improved higher LAI (Gupta et al., 2016). The contribution of biomass toward higher yield realization has earlier been reported by several studies (Slafer et al., 2009; Sadras and Lawson, 2011; Bustos et al., 2013; Aisawi et al., 2015; Gao et al., 2017) and our study further corroborate that. New cultivars in the Indian wheat breeding program have biomass gain parallel to Chinese wheat cultivars (Gao et al., 2017) (@ 62.6 kg ha^−1^ yr^−1^). The role of duration in increasing the biomass becomes more evident in early sown CA experiment where the absolute genetic gain for biomass in the varieties released after 1982 happened @ 175 kg ha^−1^ yr^−1^ and the percent variation in biomass explained by DH and DM increased significantly (Figures 9C,D).

For HI, our results are in contrast to many other studies showing that yield gain was largely because of the increase in HI with no change in biomass production (Austin et al., 1989; Sharma et al., 2012; Wu et al., 2014). Researchers also feel that probably HI has already reached a theoretical limit and a further increase in HI is not feasible (Slafer and Andrade, 1993). Our results are, therefore, clearly in agreement with the hypothesis that HI played an important role for many years after the green revolution, however an increase in biomass is mainly responsible for yield gain in recent varieties (Foulkes et al., 2009). Harvest index in Indian wheat varieties reached a maximum value of 0.419, which is well below the theoretical limit of 0.60 as proposed (Foulkes et al., 2009). The HI-value observed in our experiment under the Indian conditions has shown an almost similar increase as in many other countries like around 0.25–0.55 in China (Zhang et al., 2016; Gao et al., 2017), 0.26–0.42 in Spain (Royo et al., 2007), 0.21–0.43 in Australia (Flohr et al., 2018). In most of the varieties, the trade-off between heavy head and lodging in high biomass wheat crops along with forced maturity due to rise in temperature toward terminal stages generally led to the poor realization of HI under Indian condition like many other studies (Fischer and Edmeades, 2010; Foulkes et al., 2011; Yadav et al., 2017).

Productive tillers m^−2^ under timely sown conditions show a continuous linear improvement in spike m^2^, though at a very slow pace of 0.46 tillers per year and with comparatively larger standard error (±25.78 tillers) (Figure 4A). Similarly, GPS (Figure 4C) also showed a linear increase over years at the rate of 0.112 GPS (*p* = 0.037) with the RG of 0.245% per annum in the last 115 years. Wheat GY is largely sink-limited and yield gain particularly in winter wheat came through increased grain number against larger grain size (Shearman et al., 2005). In contrast, in spring wheat varieties of India, cubical polynomial regression improve the grain weight (Figure 4B) in recent years (*p* = 0.01). In a curvilinear relationship, the year of release accounted for 71.3% of the variation in seed weight. It is interesting to note that trend for yield component traits under early sown CA conditions is not consistent with timely sown tilled conditions for the number of spikes m^−2^ and grain weight, largely because of the conflict introduced by vernalization gene, and probably by the role of stem reserve mobilization under stress induced by a rise in temperature toward terminal stage under timely sown condition. Biomass and GY potential in wheat is largely set by stem weight and the number of tillers produced per unit area (Tausz-Posch et al., 2015) particularly under high yielding environment (Sadras and Rebetzke, 2013). The trend in tillering potential under early sowing conditions was probably thwarted by the inclusion of C 306, a high tillering variety released in 1965, and HDCSW18, a moderate tillering variety released in 2016. The strongest component for yield increase under both sets of production conditions is GPS like many other studies (Aisawi et al., 2015; Flohr et al., 2018; Liu et al., 2018) besides biomass and phenological trait. The GPS under early sown condition shows more strong linear increase than timely sown condition largely because of the increased spikelet fertility under early sown condition. The negative correlation between the number of GPS and TGW suggested as a stumbling block (Sadras and Lawson, 2011; Bustos et al., 2013) for further gain in yield was not apparent in our study as a negative correlation was offset by a prolonged vegetative phase in recent varieties supporting more number of grains and longer duration providing optimum grain-filling period. The number of GPS has increased linearly because of the increase in the number of spikelet and floret fertility, though with no change in spike length in contrast to the hypothesis proposed (Lopes et al., 2012). It indicates that both source and sink are at optimum, at least in the recently developed varieties under early sown condition. The declining linear trend for grain weight under early sown condition, however, was mainly because earlier varieties with lesser fruiting sites and enough reserve were able to fully exploit the available grain capacity to imbibe stem reserve and limitation of individual grain capacity in the recently released variety HD 3086. No strong difference in grain weight under early sown and timely sown condition in one of the recent variety like HD 3086 indicates its limitation about individual grain sink strength .Another probable reason for the declining trend for grain weight in early sown condition (Figure 6C) in contrast to timely sown can be due to prolonged stem elongation phase induced by mild vernalization requirement in some of the varieties resulted in a higher number of grains through increased number of spikelets and higher number of florets per node and interfloret competition at each node reduced the grain weight in such varieties.

The linear increase in peduncle girth under timely sown condition also supports our earlier assumption that recent varieties have probably no limitation of source and increase in biomass besides tiller number is also being contributed by increased stem girth. Similarly, PH, CL, and PL above the flag leaf has declined linearly over the years largely because of dwarfing alleles like Rht-B1b, Rht-D1b, Rht-D1c, and Rht8 being used just like another international breeding program (Zheng et al., 2011; Green et al., 2012; Lopes et al., 2012; Joudi et al., 2014; Zhang et al., 2016; Chairi et al., 2018) and their associated effect on CL, PL, and PH (Rebetzke et al., 2011, 2012) and better resource partitioning. The quadratic equation further improved the relationship (*R*^2^ = 0.7942) and it indicates an increasing trend for PH in the recently released varieties. We, therefore, assume that the biggest jump in yield gain achieved in the recent years was largely through an increase in biomass contributed by an increase in height, stem girth, and more tillering. Reduction in CL, however, introduced uncertainty in crop production because of its unsuitability for deeper seeding in early sown crop under comparatively higher mean temperature (Yadav et al., 2017) as high temperature depletes the soil moisture quickly in upper profile even under irrigated condition. Generating information on the association of other dwarfing genes with agronomically relevant traits like CL and their utilization in the breeding program can resolve these conflicts (Yadav et al., 2010), as only a limited number of dwarfing genes have been exploited in wheat breeding (Chen et al., 2015).

A very strong positive correlation of yield with DM and biomass followed by TGW, DH, and GPS and, a negative correlation with PL, PH, and CL were found. Historical studies have shown that traits, such as GPS, biomass, HI, and reduced PH are positively associated with the yield (Shearman et al., 2005; Xiao et al., 2012). The PCA divided the whole dataset into 10 principal components and the first two principal components together represent only 43.3% variation indicating the strong importance of traits explained by other principal components. DH, DM, TGW, PH, GPS, and biomass were identified through the multiple regression analysis models as highly significant traits. Based upon the multiple regression analysis, the traits were selected to establish the relationship between the average productivity of the varieties released in NWPZ since 1900 and the year of release of the variety.

**Figure 10:**
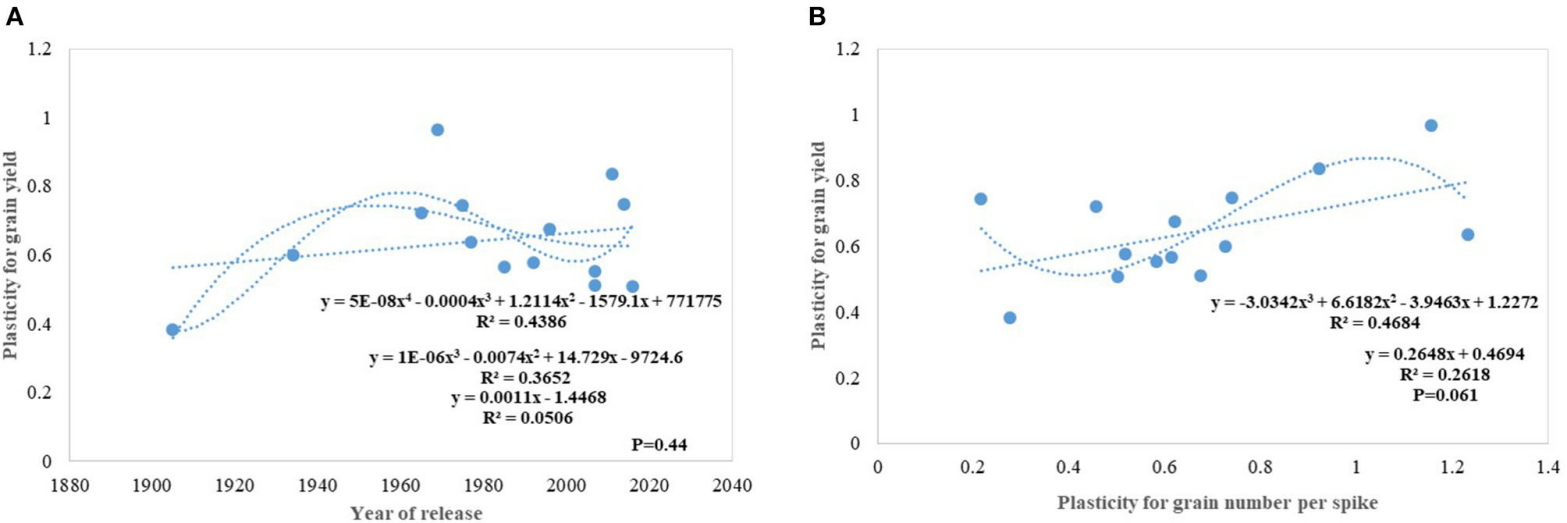
Trait plasticity for grain yield **(A)** and its linear relation with grain number **(B)** in wheat genotypes.

Under this study, we targeted to evaluate whether yield gain has any association with the competitive ability (better performance of varieties in border rows when compared to central rows) of the varieties as proposed under the ideotype breeding concept (Donald, 1981; Reynolds et al., 1994; Sadras et al., 2000; Denison, 2009) due to impaired detection and response to neighbors along with an inability to imbibe the increased available resources (Aphalo and Ballare, 1995; Ugarte et al., 2010). Response to competition was high in older tall varieties like C591 and C306, giving them more scope for weed suppression and a positive response to the availability of space. Over the years, we found no trend in RC particularly for yield and biomass; however, the component traits like GPS and tiller number show a declining trend. Polynomial regression shows that recent varieties are either neutral phenotype or with slightly higher competitive ability than their immediate predecessor for biomass production and yield. Under changing climatic conditions, it is becoming highly important that cultivars should have the plasticity to accommodate environmental variation. It indicates the ability of the varieties to respond to the improvement or deterioration in the production environment. Four out of five real mega cultivars, Kalyan Sona, PBW 343, HD 2967, and HD 3086 except HD 2329 showed comparatively higher yield plasticity, which conclusively came from different component traits among these cultivars. Polynomial regression analysis shows that an increase in yield plasticity in the recently released varieties comes largely through grain number and seed weight (Figure 10).

## Conclusion

The present study establishes the strong contribution of plant breeding activities carried out in the last 115 years in increasing the wheat productivity in the northern plains of India and there is no indication of yield plateauing in the near future. The yield gain rate has improved in the last three decades. It is very interesting to note that, the yearly gain is robust, linear, and equal to the gain achieved in other parts of the world. Under the timely sown condition, which occupies the maximum area in India, the yield gain came largely through increased crop duration with the delayed heading and biomass. Moreover, an early sown experiment showed nearly 20% higher yield gain per year largely through higher biomass and a greater number of GPS. This has proved an immense impact of phenological traits by indirectly improving the overall genetic gain in terms of many agronomic traits. In a developing country like India, where large trained manpower is available, investment in traditional plant breeding can still be highly rewarding. Though the introduction of dwarf wheat leads to a gain in productivity, the major gain comes through subsequent rounds of breeding involving these varieties and indigenous local strains in the crossing programs. The next yield jump came through Veery-derived varieties, particularly PBW 343 utilizing mild vernalization requiring genes for higher biomass production. Yield being a function of biomass and its subsequent partitioning to grain, a linear increase in HI and biomass is expected. However, our results show that HI during the last three decades has remained almost stagnant and yield gain came through improved biomass. Tiller number shows a linear increase under timely seeding condition but under early sown, it remained almost unchanged. Among the yield component, grain numbers make a contribution toward yield improvement both in the early sown and timely sown conditions. The GPS improved because of a pleiotropic effect of dwarfing gene on spike fertility (He et al., 2012) as well as due to the improved availability of photosynthate to the developing florets resulting in lesser floret abortion and its selection by the breeder (Álvaro et al., 2008). Phenological manipulation, which seems to be saturating under timely sown condition, the trend of latest varieties utilizing mild vernalization genes provide some more scope for yield gain through its manipulation under early sown condition. Modern varieties are showing either neutral behavior or a slight increase in RC and therefore more in-depth study is required to understand the role of varieties, resources, and non-resource environmental cues in modulating plant–plant interactions and their consequences for plant development and crop yield. An increase in biomass and HI happen not simultaneously but in separate phases and therefore, with a strong production of biomass in some of the recent varieties, further improvement in HI can lead to the next jump in GY in wheat. It was also proposed that yield gain can be furthered by crossing complementary varieties exhibiting high biomass and HI (Reynolds et al., 2017). We, hereby propose, in the targeted cross, further gain in yield can be realized by fully exploiting the advantage of crop duration by adjusting the time of seeding, exploring the alternate dwarfing genes that do not reduce CL, optimize the biomass production along with structural changes for lodging resistance, increasing the grain weight through optimized spike morphology without compromising grain number and increasing sugar reserve mobilization toward sink in case of a sudden rise in temperature toward the terminal stage.

## Data Availability Statement

The raw data supporting the conclusions of this article will be made available by the authors, without undue reservation.

## Author Contributions

RY and SG conceived the idea. All authors contributed to conducting the experiments and article edits. All authors contributed to the article and approved the submitted version.

## Funding

The authors would like to thank the Indian Agricultural Research Institute (IARI), New Delhi for facilitating finance to undertake the current research work.

## Conflict of Interest

The authors declare that the research was conducted in the absence of any commercial or financial relationships that could be construed as a potential conflict of interest.

## Publisher's Note

All claims expressed in this article are solely those of the authors and do not necessarily represent those of their affiliated organizations, or those of the publisher, the editors and the reviewers. Any product that may be evaluated in this article, or claim that may be made by its manufacturer, is not guaranteed or endorsed by the publisher.
