# Supplementary material for: Genetic Gain in Yield and Associated Changes in Agronomic Traits in Wheat Cultivars Developed Between 1900 and 2016 for Irrigated Ecosystems of Northwestern Plain Zone of India
Source: Front Plant Sci. 2021 Sep 23;12:719394. doi: 10.3389/fpls.2021.719394 (PMC8496457; doi:10.3389/fpls.2021.719394)
Supplement: Supplementary file 1 [file Data_Sheet_1.docx]

**Supplementary Table 1. Analysis of variance for yield and its contributing traits**

| **Source** | **Varieties** | **Year** | **Varieties × year** | **Breeding period** | **Breeding Period × Year** | **Genotypes within breeding period** |
| --- | --- | --- | --- | --- | --- | --- |
| **DF** | **13** | **3** | **39** | **6** | **18** | **7** |
| **DH** | 162.2*** | 911.1*** | 18.7*** | 267.3*** | 15 | 72.13 |
| **DM** | 186.68*** | 156.53*** | 13.15*** | 254.67*** | 23.38 | 128.4*** |
| **PH** | 852*** | 132.6** | 35.7* | 1761.5*** | 59.6** | 72.5* |
| **CL** | 0.795*** | 0.0224 | 0.2706*** | 1.4037*** | 0.471*** | 0.2686** |
| **Tillers per plant** | 3762 | 40256*** | 3429 | 2025 | 5186* | 5251 |
| **Biomass** | 12288677*** | 3616119* | 2201276** | 22981347*** | 3753850*** | 3123532 |
| **SPS** | 14.224*** | 4.063* | 2.178* | 15.903*** | 2.643 | 12.785 |
| **GPS** | 332.9*** | 29.6 | 37* | 471.4*** | 38.8 | 214.1*** |
| **TGW** | 140.75*** | 92.67*** | 5.62 | 188.8*** | 6.58 | 99.56*** |
| **Grain Yield** | 5332471*** | 7895094*** | 242340 | 11315871*** | 282847 | 203842 |

**Supplementary Table 2. AMMI analysis for grain yield**

| **Source** | **Df** | **Sum Sq** | **Mean Sq** | **F value** | **Pr(>F)** | **Proportion** | **Accumulated** |
| --- | --- | --- | --- | --- | --- | --- | --- |
| ENV | 3 | 23685282 | 7895094 | 35.68909 | 2.40E-03 | - | - |
| REP (ENV) | 4 | 884874.7 | 221218.7 | 1.23416 | 3.08E-01 | - | - |
| GEN | 13 | 69322117 | 5332471 | 29.7494 | 1.70E-19 | - | - |
| GEN:ENV | 39 | 9451254 | 242339.8 | 1.351993 | 1.54E-01 | - | - |
| PC1 | 15 | 5557786 | 370519.1 | 2.07 | 2.72E-02 | 58.8 | 58.8 |
| PC2 | 13 | 2707744 | 208288 | 1.16 | 3.34E-01 | 28.6 | 87.5 |
| PC3 | 11 | 1185724 | 107793.1 | 0.6 | 8.20E-01 | 12.5 | 100 |
| Residuals | 52 | 9320810 | 179246.4 | - | - | - | - |
| Total | 150 | 1.22E+08 | 814103.9 | - | - | - | - |

**Supplementary Table 3. Summary of stepwise regression analysis without breeding period**

| **Stepwise Summary without Breeding period** | | | | | | |
| --- | --- | --- | --- | --- | --- | --- |
| **Variable** | **Method** | **AIC** | **RSS** | **Sum Sq** | **R-Sq** | **Adj. R-Sq** |
| DM | addition | 1827.468 | 75809361 | 36854977 | 0.32712 | 0.321 |
| Biomass | addition | 1800.18 | 58365283 | 54299055 | 0.48195 | 0.47245 |
| TGW | addition | 1790.854 | 52751595 | 59912743 | 0.53178 | 0.51877 |
| CL | addition | 1777.506 | 45996033 | 66668305 | 0.59174 | 0.57648 |
| GPS | addition | 1770.993 | 42629715 | 70034623 | 0.62162 | 0.60377 |
| PH | addition | 1767.407 | 40555427 | 72108911 | 0.64003 | 0.61946 |
| EL | addition | 1766.076 | 39367268 | 73297070 | 0.65058 | 0.62706 |
